# Infection prevention and chronic disease management in cystic fibrosis and noncystic fibrosis bronchiectasis

**DOI:** 10.1177/1753466620905272

**Published:** 2020-03-11

**Authors:** Sherstin T. Lommatzsch

**Affiliations:** National Jewish Health, 1400 Jackson Street, Denver, CO 80206-2762, USA

**Keywords:** bronchiectasis, chemoprophylaxis, cystic fibrosis, eradication, immunoprophylaxis, infection control, infection prevention, pulmonary exacerbation, resection

## Abstract

Bronchiectasis is a chronic lung disease (CLD) characterized by irreversible bronchial dilatation noted on computed tomography associated with chronic cough, ongoing viscid sputum production, and recurrent pulmonary infections. Patients with bronchiectasis can be classified into two groups: those with cystic fibrosis and those without cystic fibrosis. Individuals with either cystic fibrosis related bronchiectasis (CFRB) or noncystic fibrosis related bronchiectasis (NCFRB) experience continuous airway inflammation and suffer airway architectural changes that foster the acquisition of a unique polymicrobial community. The presence of microorganisms increases airway inflammation, triggers pulmonary exacerbations (PEx), reduces quality of life (QOL), and, in some cases, is an independent risk factor for increased mortality. As there is no cure for either condition, prevention and control of infection is paramount. Such an undertaking incorporates patient/family and healthcare team education, immunoprophylaxis, microorganism source control, antimicrobial chemoprophylaxis, organism eradication, daily pulmonary disease management, and, in some cases, thoracic surgery. This review is a summary of recommendations aimed to thwart patient acquisition of pathologic organisms, and those therapies known to mitigate the effects of chronic airway infection. A thorough discussion of airway clearance techniques and treatment of or screening for nontuberculous mycobacteria (NTM) is beyond the scope of this discussion.

## Introduction

Bronchiectasis is a chronic lung disease (CLD) characterized by irreversible bronchial dilatation noted on computed tomography with associated chronic cough, ongoing viscid sputum production, and recurrent pulmonary infections.^1^ Patients can be classified into two groups: those with cystic fibrosis (CF) and those without CF. Individuals with either CF-related bronchiectasis (CFRB) or non-CF-related bronchiectasis (NCFRB) experience continuous airway inflammation leading to airway changes fostering development of a unique polymicrobial community. Chronic pulmonary infection with many of these organisms worsens airway inflammation, triggers pulmonary exacerbations (PEx), reduces quality of life (QOL), and, in some cases, is an independent risk factor for increased mortality. As there is no cure for either condition, prevention and control of infection is paramount.

Infection prevention is a complex undertaking aimed to reduce acquisition of pathologic organisms and mitigate their effects once a patient becomes infected. More specifically it incorporates transmission prevention, immunoprophylaxis, and, occasionally, antimicrobial prophylaxis.^2–4^ If acquired, however, some organisms may be eradicated from the airway.^5^ Should eradication be unsuccessful, chronic suppression of inflammation and these organisms’ activity becomes the focus of therapy. Topics important to infection control and prevention, but are beyond the scope of this discussion, are details regarding mechanical airway clearance and treatment of, or screening for, nontuberculous mycobacteria (NTM).

## Transmission prevention: education, immunoprophylaxis, organism source containment

Transmission prevention and control is a complex stratagem aimed to reduce the likelihood that a patient will acquire a pathologic organism or transmit that organism to another individual. Multiple recommendations have been published by the United States (US) Centers for Disease Control (CDC), Cystic Fibrosis Foundation (CF Foundation), World Health Organization (WHO), and other professional societies relevant to patients with either CFRB or NCFRB.^4^ Overall, it is a multi-faceted endeavor incorporating the education of patients, their family, and healthcare team; immunoprophylaxis; and pathogen source containment. The following sections describe these strategies.

### Education

Team education is a sizable undertaking, incorporating the rationale for infection prevention with the methods by which microbial transmission is best prevented.^4,6^ To affect change, all patients, family members, and healthcare team should be included in this endeavor.^4^ It is important to remember that the healthcare team is not limited to clinical providers, it includes anyone who may have contact with a patient, such as scheduling staff, phlebotomists, radiology technicians, and environmental service providers.

A clinic or hospital ward needs a clinical champion tasked with organizing education plans and ensuring compliance with established protocols. Teaching materials and presentations should be tailored to the recipients’ education level, age, pre-existing conceptions regarding prevention, and their specific role on the team. Patients must be empowered to ask their providers to follow known guidelines, and team members need to educate colleagues when a lapse is noticed. Regular training of personnel, and immediate individual feedback following a protocol deviation, help provide effective institution of protocols and improves individual clinical practice. Finally, as the quality of feedback is strongly related to guideline adherence, a formal review process outlining team successes and areas needing improvement should occur every 3–6 months.^4,6^

### Immunoprophylaxis

#### CFRB and NCFRB

Immunoprophylaxis prevents disease *via* passive or active immunity.^7^ Unfortunately for both adults and children, there is global discordance between recommended vaccination schedules for those with CLD.^8^ Additionally, CFRB and many causes of NCFRB lead to systemic diseases that may benefit from nonpulmonary vaccination. Thus, even though it is recommended that persons with CLD receive pulmonary vaccinations according to a nation’s recommended schedule, one must also consider nonpulmonary organ involvement to determine which of all available vaccines may be beneficial for patients.

The CDC has multiple suggestions regarding vaccination for persons with CLD.^9^ Foremostly, this should include the annual influenza vaccine, which is recommended for all persons 6 months of age and older (unless a medical contraindication exists).^10^ These persons should also receive standard treatment or chemoprophylaxis with oseltamivir if diagnosed with, or exposed to, acute influenza A or B.^9,11^ In contrast with healthy persons, those with CLD are recommended to receive pneumococcal vaccination beginning much earlier than healthy individuals; at age 19 instead of 65.^9^ Important to keep up-to-date is the pertussis booster vaccination.^3,9^ In CFRB, however, practice patterns differ sometimes differ from these guidelines (see below).

As liver disease complicates the clinical course in many patients with CFRB or NCFRB, one could consider viral hepatitis vaccination in these patients.^3,12^ For example, 3.4% of all US patients with CF have liver disease,^13^ as do 40% of those with Pi ZZ alpha-1- antitrypsin deficiency.^14^ Persons with CF experience noncirrhotic or cirrhotic liver disease in 3.9% and 2.6%, respectively, and up to 17% of children with CF have clinically significant liver disease.^15,16^ Of persons with alpha-1 antitrypsin deficiency (Pi ZZ), 40% have histologic evidence of significant liver disease or frank cirrhosis.^14^ Despite the inactivated hepatitis A and recombinant hepatitis B vaccinations having been shown to be safe and effective in those with chronic liver disease, there is a paucity of recommendations aimed to capture those inadequately vaccinated.^3,14–26^

#### CFRB

Historically, *Streptococcus pneumoniae* has not been commonly cultured from CF sputa.^15^ Some have speculated that its isolation is underestimated due to the dominance of other bacteria.^3^ Additionally, recent publications suggest a pediatric carrier state of 4.8–7.4% to as high as 12.7–28.6%.^17,18^ Nevertheless, there is little data demonstrating invasive pneumococcal disease in CF, thus the clinical relevance of pneumococcal vaccination seems to be unclear.^19^ On the other hand, the risk of both pulmonary and invasive pneumococcal disease significantly increases following lung transplantation.^20,21^ Thus, as immunosuppression blunts immune responsiveness, pretransplant pneumococcal vaccination is important.^3^ Additionally, the long-term impact of the new CF Transmembrane Regulator Protein modulators (ivacaftor, lumacaftor/ivacaftor, tezacaftor/ivacaftor, elexacaftor/tezacaftor/ivacaftor, and those currently in development) on the life expectancy and pulmonary microbiota of those with CFRB is unknown, which should prompt caregivers to consider whether these vaccinations may be indicated.

### Organism source containment

#### CFRB and NCFRB

Source containment of would-be pathogens comprises both the understanding of how organisms are transmitted and the precautions required to diminish their spread. In practice, it addresses how those with bronchiectasis interact with their environment.^4^ Individuals acquire new flora from person-to-person contact, contaminated medical equipment, and *via* a multitude of ecological sources.^22^

Person-to-person transmission may occur from direct contact, indirect contact, droplet transmission, and airborne transmission of droplet-nuclei (Table 1).

**Table 1.** Methods of person-to-person transmission.

Type	Definition	Organism examples
Direct contact	Immediate exposure to contaminated secretions (kissing)	MRSA *Pseudomonas aeruginosa, Burkholderia* spp. RSV
Indirect contact	Exposure *via* a hard surface or intermediary object (hand, toy, door handle, countertop, medical equipment)	Same as direct contact
Droplet	Aerosolized material >5 µm which may travel 1–2 m from its source and infect *via* direct deposition onto mucous membranes	MRSA *Pseudomonas aeruginosa* Burkholderia spp. Mycoplasma pneumoniae Bordetella pertussis Rhinovirus Influenza virus Adenovirus RSV
Airborne (droplet nuclei)	Aerosolized material ⩽5 µm^a^ that remains suspended in air for an extended time and is inhaled into the lower respiratory tract	*Mycobacterium Tuberculosis* Measles Varicella-zoster SARS-CoV

Adapted from Table 5 in ‘Infection and Prevention Control Guideline for Cystic Fibrosis: 2013 Update’.^4^

a Some sources define a droplet nucleus as <3.3 µm.^4^

MRSA, methicillin-resistant *Staphylococcus aureus*, RSV, respiratory syncytial virus; SARS-CoV, severe acute respiratory syndrome coronavirus.

Patients expel these infectious particles when they cough, sneeze, speak, undergo chest physiotherapy (CPT), perform pulmonary function testing (PFTs), or are intubated or suctioned.^4^

Most organisms are transmitted *via* one primary mode, but transmission may occur *via* other routes. *Pseudomonas aeruginosa*, for example, is typically acquired from water sources (reservoirs, hot tubs, sinks, and showers). However, as it survives longer in CF sputum, it may be transmitted *via* a handshake up to 180 min following skin contamination.^23^ It has been isolated from infectious droplets in hospital rooms, clinic hallways, and following pulmonary function tests 45–120 min after an infected individual has departed.^4^ Additionally, infectious droplets were implicated as the vector by which an epidemic outbreak occurred at a European adult CF center.^24^ Methicillin resistant *Staphylococcus aureus* (MRSA) may spread *via* contact or infectious aerosols.^4^ Influenza is usually transmitted *via* fomites or droplets, but some suggest that airborne isolation should be implemented in pandemic situations.^4,25–27^

In addition to incorporating recommendations to decrease cross-infection, as outlined by the CDC and WHO, many outpatient clinics incorporate cohort segregation (i.e. patients with *Burkholderia spp.* being seen on one day and those with *P. aeruginosa* on another) or physical separation by distance into their infection control practice.^28^ Several studies have reported reductions in the rate of patient-to patient transmission of *P. aeruginosa* and *Burkholderia spp.* by combining cohort segregation with other interventions such as equipment decontamination, patient education, and use of barrier precautions.^28,29^ As there is little data on cohort segregation alone (i.e. without being combined with other infection control interventions), the CF Foundation supports only physical separation.^4^ Currently, physical separation is defined as approximately 2 m.^4,22^ Recent data, however, suggests that infected droplet nuclei may travel as far as 4 m^30^; This is an important consideration in clinic as patients wait in lines and lobbies from check-in to check-out. Finally, regardless of the healthcare setting, all patients should wear a mask when not in an exam or hospital room.^4^

Healthcare workers (HCW) need to practice meticulous hand hygiene and use appropriate personal protective equipment (PPE). They should clean their hands before and after contact with patients using a waterless antiseptic cleanser as these are superior to antimicrobial soap.^4,22^ Upon exiting from a patient room isolated for *Clostridium difficile*, hand cleansing using soap and water is preferred as alcohol-based cleaners do not disinfect *C. difficile* spores.^31,32^ Those having contact with patients or their expelled airway secretions should don gown and gloves. When relevant, team members may need to wear a mask (surgical or N-95) or protective eye covering (intubation).^4,26,33,34^ Providers must clean their stethoscopes between patients or use patient-specific equipment.^4,26,33,34^

**Figure 1.**
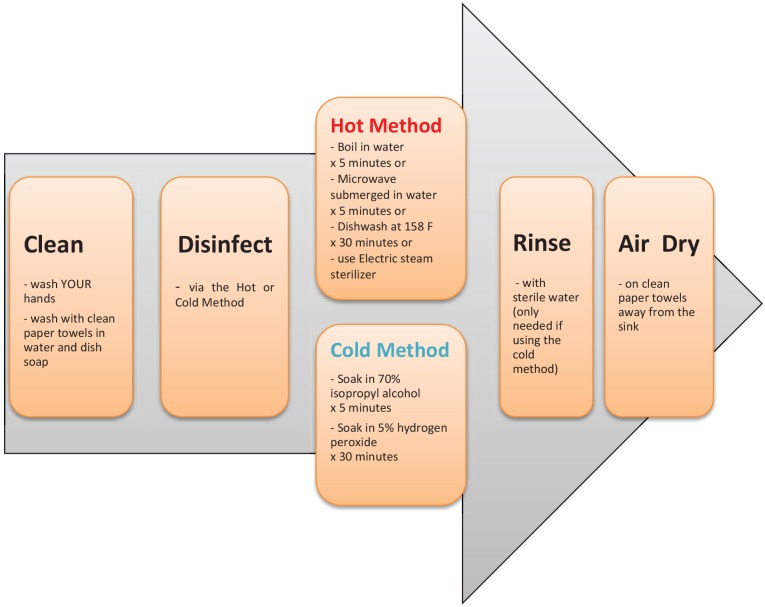
Nebulizer disinfection: hot and cold methods.^4,35^ Neither the isopropyl alcohol nor hydrogen peroxide should be used a second time.

Pulmonary function tests (PFTs) result in the production of many aerosols. Whether patients are completing basic spirometry or complete pulmonary testing, the procedure must be performed in a negative pressure room or one equipped with a high efficiency particulate air (HEPA) filter. If this precaution is not possible, then each test should be separated by 30 min.^4^ Methodical cleaning of medical equipment is paramount to infection control. Airway clearance devices, nasal irrigation devices, and spacers may be cleaned with tap, well, distilled, or bottled water when followed by disinfection.^4,35^ Nebulizers should be maintained using either the hot or cold method (Figure 1), incorporating machine specific instructions as outlined by the manufacturer.^35^

Respiratory equipment and humidifier reservoirs need to be cleaned and used with sterile water.^4,35^

To avoid acquisition of soil-borne organisms such as *Burkholderia* spp. (typically a CF pathogen) and *Aspergillus* spp., the CF Foundation recommends minimal exposure to activities generating dust from organic matter.^4^ Hot tubs or stagnant water should be avoided as *P. aeruginosa* is often harbored in these environments.^4^

## Pulmonary microbiota, microbial chemoprophylaxis, eradication, chronic pulmonary therapy, and thoracic surgery

Quotidian pulmonary care is essential in both CFRB and NCFRB to control the effects of mucosal inflammation and chronic microbial infection. Moreover, in CFRB, infection with *P. aeruginosa* and MRSA are associated with higher mortality, accelerated loss of lung function, more frequent exacerbations, increased number of hospitalizations, and diminished QOL. *P. aeruginosa*, but not *S. aureus* (including MRSA), has similar consequences in NCFRB.^36–38^ Thus, most treatments are centered around the specific pulmonary microbiota of a patient’s lungs. To prevent or delay such outcomes, patients may be managed with oral antimicrobial primary chemoprophylaxis, bacterial eradication attempts, targeted inhaled antibacterial agents, thoracic surgery, and inflammation control. Despite the symptomatic similarities between CFRB and NCFRB, not all therapies are effective in both groups, and, in NCFRB, many therapies have not yet been studied. To clearly differentiate between recommendations for these different populations, each will be discussed within the classification of CFRB or NCFRB.

### CFRB

#### Pulmonary microbiota

**Figure 2.**
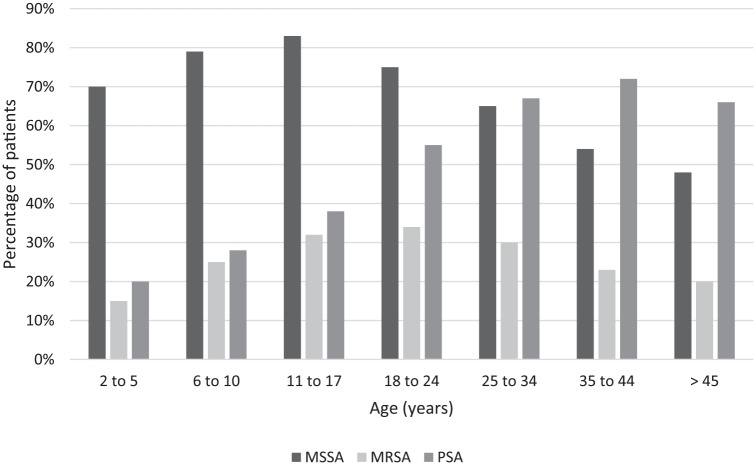
Age-based CF pulmonary microbiota. Adapted from CF Foundation Registry 2016; Cystic Fibrosis Foundation Patient Registry 2016 Annual Data Report Bethesda, MD, USA. Other gram-negative rods not shown. CF, cystic fibrosis.

Children with CF (ages birth to 17 years) are initially infected with methicillin sensitive *S. aureus* (MSSA) (60–70%), followed by *Haemophilus influenza* peaking at 30% in school-aged children, and then *P. aeruginosa*. The presence of *P. aeruginosa* rises steadily through adolescence and becomes the most prominent organism infecting the CF airway in adulthood. Other important organisms in adults include MSSA, MRSA, multidrug resistant Pseudomonas, and other gram negative rods such as *Burkholderia cepacia complex* (not shown) (Figure 2).^15^

#### Primary chemoprophylaxis

Primary antimicrobial chemoprophylaxis for MSSA is controversial within the CF community. Commonly practiced in Europe, children from the age of diagnosis through 2 years may be given daily flucloxacillin as recommended by the United Kingdom (UK) based CF Trust.^39^ Published literature shows that CF children have fewer isolates of MSSA, less cough and reduced hospitalizations when a narrow spectrum antistaphylococcal antibiotic, such as flucloxacillin, is started in early infancy and continued through 6 years of age.^40^ This practice does not result in lung function improvement.^39,40^ Studies using prophylactic cephalexin resulted in an increased risk of *P. aeruginosa* infection,^41,42^ and a 2017 Cochrane review noted a trend towards increased *P. aeruginosa* isolation in children aged 4–6 years treated with flucloxacillin.^40^ Due to concerns regarding accelerated *P. aeruginosa* acquisition, some American pediatric pulmonologists remain reticent to implement this practice, and, currently, the CF Foundation recommends against its use.^43^ To better define long-term flucloxacillin treatment in infants with CF, there is an ongoing UK study: ‘The cystic fibrosis (CF) anti-staphylococcal antibiotic prophylaxis trial (CF START); a randomized registry trial to assess the safety and efficacy of flucloxacillin as a long-term prophylaxis agent’ (ISRCTN18130649 https://doi.org/10.1186/ISRCTN18130649).

#### Eradication

An organism’s pathogenicity refers to its ability to increase PEx frequency, diminish lung function and increase mortality.^4^ As bacteria increase airway inflammation, the rationale to eradicate is to diminish the long-term pulmonary consequences associated with ongoing inflammation by preventing chronic infection.^4^ Both the UK CF Trust and European Cystic Fibrosis Society (ECFS) recommend eradication of *P. aeruginosa*, MSSA, MRSA, and *H. influenzae*,^22,39,44^ whereas the CF Foundation has specific eradication recommendations only for *P. aeruginosa*.^45^ Due to its devastating effects on lung function, and being a relative contraindication to lung transplantation, regimens to eliminate *B. cepacia complex* are currently being studied, with reports of success beginning to appear in the literature.^46–48^

*Pseudomonas aeruginosa*. Eradication of *P. aeruginosa* in CFRB may be accomplished with inhaled tobramycin solution or dry powder.^45,49,50^ Alternatively, inhaled aztreonam lysine may be used.^51^ Its use is not specifically discussed in the most recent CF Foundation guidelines, but the Foundation noted that data supporting inhaled aztreonam had not yet been released when its recommendations were made. In Europe, as strongly recommended by the CF Trust, patients are often started on inhaled colistin with oral ciprofloxacin.^39^

*Methicillin sensitive Staphylococcus aureus*.^39^ For MSSA-culture-positive CF patients, the CF Trust and ESCF recommend oral therapy for 2–4 weeks. If already on primary chemoprophylaxis with flucloxacillin, the dose of flucloxacillin is increased and a second anti-staphylococcal antibiotic added. If eradicated and not on daily prophylaxis, 2–4 weeks of treatment every time a patient is culture positive may be continued. Alternatively, the CF Trust suggests that regardless of preculture therapy, the patient be continued on secondary chemoprophylaxis. The duration of secondary chemoprophylaxis is unclear. For further details, the reader is referred to the CF Trust Antibiotic Treatment in Cystic Fibrosis 3rd Edition.^39^ Eradication is not specifically reviewed by the CF Foundation, but should a patient become chronically infected with MSSA, the Foundation states there is insufficient evidence to support or recommend against secondary chemoprophylaxis.^43^

*Haemophilus influenza.*^39^ To eradicate *H. influenzae*, the UK CF Trust and ESCF recommend *H. influenzae* directed oral therapy for 2–4 weeks.^22,39^ Should *H. influenzae* persist in the sputa, antibiotic therapy is continued and the clinician may consider continuous suppressive antibiotics as is done for MSSA. The CF Foundation has no current recommendations regarding eradication or secondary prophylaxis for *H. influenzae*.

*Methicillin resistant Staphylococcus aureus*. There is no global consensus regarding an effective MRSA eradication protocol. Eradication is recommended by the UK CF Trust, and the effectiveness of different protocols for acute and chronic infection is being actively studied.^39^ The UK CF Trust suggests patients be given rifampicin and fusidic acid, with or without inhaled vancomycin, but there is little data to support this regimen.^52^ The Northern Ireland Regional Adult Cystic Fibrosis Center in Belfast published a retrospective review of 37 patients with newly acquired MRSA. The majority of patients were treated using the CF Trust’s oral regimen (i.e. without inhaled vancomycin) with added 2% nasal mupirocin and 4% chlorhexidine body wash for 5 days. This resulted in a 79% success rate, but, not unexpectedly, was complicated by known fusidic acid gastrointestinal distress in many patients.^52^ In the US, Johns Hopkins University recently published results of a 28-day phase II trial in chronic MRSA pulmonary infection evaluating the combination of oral therapy (rifampin combined with either trimethoprim-sulfamethoxazole or doxycycline) and skin treatment (with mupirocin nasal cream and 4% chlorhexidine body wash) with or without inhaled vancomycin (NCT01594827). They did not find any significant difference in sputum culture at 1 and 3 months between those receiving inhaled vancomycin and those who received taste matched placebo.^53^ Additionally, the START-too study team is actively recruiting patients for an open-label phase II study to examine the effect of oral antibiotics (trimethoprim-sulfamethoxazole or minocycline), intranasal mupirocin and 0.12% chlorhexidine to gargle on the microbiota of patients with newly acquired MRSA pulmonary infection (NCT03489629).

#### Chronic pulmonary therapy

Chronic pulmonary therapy is a daily (or multiple times daily) medical regimen aimed to improve clinical outcomes by improving a patient’s mucociliary clearance. This is a multi-faceted approach utilizing airway clearance techniques (ACT) combined with inhaled osmotic and mucolytic aerosols, antimicrobial agents, and anti-inflammatory medications.^54^

A variety of ACT are utilized in both CFRB and NCFRB. The purpose is to loosen viscous mucous from airway walls utilizing mechanical force. These include: CPT with or without positional drainage, huff coughing, positive end expiratory pressure with or without oscillation, autogenic drainage, and high frequency chest oscillation. These methods improve patient symptoms and QOL scores, but none has yet been shown to be superior to another in either disease state.^54–57^ These maneuvers are typically used simultaneously with nebulized medications. For more detailed information regarding current studies and recommendations, the reader is referred to Wilson LM and Colleagues-Cochrane Review 2019, Flume PA and Colleagues-Respiratory Care 2009, and Lee AL and Colleagues-Cochrane Review 2017.

#### Aerosolized osmotic and mucolytic agents

Recommendations for aerosolized osmotic and mucolytic agents is often based on disease severity as defined by percent predicted Forced Expiratory Volume in 1 s (ppFEV_1_): Normal, greater than 90%; mildly impaired, 70–89% predicted; moderately impaired, 40–69% predicted; and severely impaired, 40% predicted.^58^ They function by improving the quality of the airway surface liquid (ASL). Additionally, as these agents may cause bronchospasm, one should consider a tolerance test prior to initiating therapy or pretreat their use with a short acting beta-agonist.^43,50^

The lung defends itself against inhaled pathogens primarily *via* mucociliary clearance, a vital part of which is healthy ASL. ASL has been well studied in CF. Based on a gel on mucous model, ASL is comprised of two layers; the perimembranal aqueous layer, termed periciliary liquid (PCL), over which lies a mucous layer intended to entrap inhaled pathogens.^59,60^ Ideally, the PCL is the height of an extended cilium (approximately 7 µm), allowing the cilia to beat unimpeded in a thin liquid layer. The cilia propel thicker mucous layer is caudally separated from the mucin rich mucous.^59,60^ In CFRB, both layers lose volume (thus height) due to dehydration, resulting in viscous mucous plaques and plugs containing neutrophils, entrapped microorganisms, and other particulate matter.^59–61^

Aerosolized osmotic agents (also known as hydrators) are intended to thin the ASL. Both hypertonic saline (HTS) and dry powder mannitol have been studied for this purpose. Inhaled 7% hypertonic saline results in a sustained increase mucous clearance, a small absolute difference in lung function, mild improvement in QOL, and reduction of PEx in patients with a ppFEV_1_ of >40–50%.^62–64^ It is thus recommended for use in CF patients older than 6 years by the CF Foundation, and is part of the ECFS Best Practice Guidelines.^43,50^ Dry powder mannitol has been used in Europe for some time. It is recommended by the ECFS as it increases FEV_1_, and may reduce the number of PEx in patients with moderately impaired lung disease.^50,65,66^ In May 2019, the US Federal Drug Administration approved its use to improve lung function in CF adults based on the results from the Long Term Administration of Inhaled Mannitol in Cystic Fibrosis – A Safety and Efficacy Trial in Adult Cystic Fibrosis Subjects. This double-blinded, randomized, parallel, multicenter study randomized 350 CF adults to receive either inhaled mannitol 400 mg twice daily or placebo twice daily for 26 weeks. Patients in the study arm had a significant increase in FEV_1_ compared with placebo. Both groups, however, had a similar number of PEx and adverse events (NCT02134353).

Extracellular DNA is released by leukocytes and constitutes approximately 10% of the dry weight of CF airway secretions.^67^ Recombinant human DNase (rhDNase) also known as dornase-alpha, hydrolyzes DNA, and, in so doing, decreases the viscosity of the ASL in patient with CF. With chronic use (at least 12–26 weeks), rhDNase has been has been shown to improve QOL in patients with moderate-to-severe lung disease as well as reduce PEx frequency and increase ppFEV_1_ in patients with mild to severe lung disease.^67,68^ These results are not sustained with discontinuation, and, thus, the CF Foundation and ECFS recommend long-term use.^43,50^ Another mucolytic agent, N-acetylcysteine, has been trialed but has not been found to have a beneficial effect on pulmonary function in CFRB.^69^

#### Antimicrobial agents

Treatment with inhaled antibiotics is recommended for patients with CFRB chronically infected with *P. aeruginosa.*^43^ CF patients with chronic pseudomonal infection are prescribed a cycling 28-day on/off regimen of inhaled antibiotic therapy. Tobramycin twice daily (as solution or dry powder) or aerosolized aztreonam three times daily may be prescribed.^49,50^ Inhaled colistin solution or dry powder is recommended by the ECFS and the CF Trust, but its use not yet recommended by the CF Foundation.^39,43,50^

In CF patients chronically infected with *P. aeruginosa*, both inhaled tobramycin and enteral macrolide therapy reduce exacerbation frequency and loss of ppFEV_1._^70,71^ The 2003 azithromycin trial published in the *Journal of the American Medical Association* (JAMA) included a balanced number of patients on chronic inhaled tobramycin in the intervention and control groups. This suggested that azithromycin has an added benefit to inhaled tobramycin,^70^ and the use of both is recommended by the CF Foundation, CF Trust and ECFS.^39,43,72^ Over the past several years, however, new data suggests that the concomitant use of inhaled tobramycin and oral azithromycin may have an antagonistic effect. In 2017, Nichols and colleagues published in the *Journal of Cystic Fibrosis* that there was no change in ppFEV_1_, but a decreased QOL when tobramycin and azithromycin were used together. Additionally, there was a greater decrease in the bacterial burden of *P. aeruginosa* when inhaled tobramycin was used alone *versus* the combination. Interestingly there was an increase in ppFEV_1_ and QOL in patients using azithromycin with inhaled aztreonam.^73^ To further study the relationship between inhaled tobramycin and enteral azithromycin, Seattle Children’s Hospital is sponsoring a phase IV prospective, randomized, double-blinded, placebo-controlled trial of the addition of thrice weekly azithromycin 500 mg to chronic inhaled tobramycin in adolescents and adults with CF (NCT02677701).

At this time, the CF Foundation does not recommend chronic suppression with inhaled antibiotics for any other organism due to a paucity of data. Currently, however, Savara Pharmaceuticals is conducting a phase III double blind, multi-center, randomized placebo controlled parallel-group study analyzing the mean absolute change in ppFEV_1_ in patients given 30 mg of inhaled vancomycin powder twice daily (NCT03181932).

#### Anti-inflammatory medications

Although traditionally used as an antimicrobial, azithromycin is used as an anti-inflammatory agent in CFRB. Continual azithromycin was initially recommended for those chronically infected with *P. aeruginosa*, and it remains so by the CF Foundation.^43^ Over the past several years, accumulating data suggests that patients not chronically infected with pseudomonas may also benefit from ongoing macrolide therapy. A 2012 Cochrane review included CF patients regardless of pseudomonal status, and found that azithromycin reduces the frequency of pulmonary exacerbation and improves lung function. The authors mention that, although pseudomonal status is unlikely a confounding factor regarding pulmonary function, further studies in those not infected with this organism are needed.^74^ Currently, both the CF Foundation and CF Trust suggest consideration of macrolide therapy in those not chronically infected with pseudomonas.^39,43^ Regardless of the population in which azithromycin is being used, each patient must be screened for NTM infection prior to beginning therapy, and throughout the course of its use.^39,43^ One should monitor for side-effects such as QT prolongation, ototoxicity, and nephrotoxicity.^1,39^

Inhaled corticosteroids are not recommended as chronic therapy.^43^ These medications are indicated for use only if patients have concomitant asthma or chronic obstructive pulmonary disease.^43^ The use of HMG Co-A reductase inhibitors has not yet been adequately studied in the CF population. Ibuprofen has been studied in CFRB, and is efficacious in the pediatric population, but there is insufficient data to recommend for or against its use in adults.^43^ Finally, leukotriene modifiers require further study before they are recommended for chronic use as an anti-inflammatory.^43^

#### Thoracic surgery

The role of thoracic surgery in patients with CFRB is typically reserved for recurrent or refractory infections, hemoptysis isolated to a given lobe or lobar segment, and refractory pneumothorax.^75–78^ Surgery should be undertaken only following a discussion of the relative merits of the intended operation, patient QOL without the operation, and the future need for lung transplantation. As surgery is performed infrequently, available data are often weakened by retrospective design or small sample size. The available analyses, however, have reported mixed conclusions, often due to differences in patient age and severity of lung disease as measured by ppFEV_1_. In their cohort of 10 pediatric patients (ages 2¾–19 years) Marmon and colleagues reported improved symptomatology, fewer subsequent hospitalizations, and no need for postoperative tracheostomy.^76^ Their population had a ppFEV_1_ predominately ⩾60% and their two reported deaths had a pre-operative ppFEV_1_ in this range. Smith and colleagues reported a similar clinical and functional experience in 14 patients ages 9¾–20 years old.^79^ However, they noted that patients with a ppFEV_1_ < 30% had a poor outcome. This finding was corroborated by Sheikh and colleagues in their population of 15 patients (median age 20.6 ± 10.5 years), but in patients with a ppFEV_1_ of ⩽40%.^75^ Many individuals in each of these three groups also experienced a postoperative decline in ppFEV_1_. Furthermore, although long-term mortality is not affected, patients transplanted for any indication with a history of prior cardiothoracic surgery (CTS) have lower post-transplant peak ppFEV_1_, higher rates of respiratory and renal complications, and increased postoperative bleeding when compared with those without prior CTS.^80^ Thus, there remains a precarious balance between patients’ current and possible future treatment.

### NCFRB

#### Pulmonary microbiota

Interestingly, despite the similarities of patient signs and symptoms, the microbiota of CFRB and NCFRB are different. The true prevalence of specific organisms infecting the NCFRB airway is difficult to describe as there is a paucity of pooled data. Culture data reported from different studies and those from the recently published US Bronchiectasis Registry Report reveal a wide distribution of several prominent organisms: *P. aeruginosa*, community acquired organisms such as *H. influenza* and *Moraxella catarrhalis*, and anaerobic organisms (Table 2).^81–84^ Note that NTM was very common in the Bronchiectasis Registry Report (30%), but this may be secondary to a registration bias from a large NTM treatment center actively enrolling in the registry.^84^

**Table 2.** Reported NCFRB microbiota.

Common organisms	Percentages reported^81–84^
*Haemophilus influenzae*	8–52%
*Pseudomonas aeruginosa*	9–43%
*Moraxella catarrhalis*	1–27%
*Streptococcus pneumoniae*	3–37%
*Enterobacteriaceae*	7%
NTM	30%
*MSSA*	3–27%
*MRSA*	2–3%
*Oropharyngeal flora*	74%
*Prevotella*	45%
*Veillonella*	33%

NCFRB, noncystic fibrosis related bronchiectasis; MRSA, methicillin-resistant *Staphylococcus aureus*; MSSA, methicillin-sensitive *Staphylococcus aureus*; NTM, nontuberculous mycobacteria.

#### Primary chemoprophylaxis

There are no specific recommendations for primary chemoprophylaxis for patients with NCFRB.

#### Eradication

Unlike CFRB, recommendations for bacterial eradication in NCFRB are published only for *P. aeruginosa* as there is a lack of data evaluating regimens for other organisms.^1,39,50^ Please note that the recommended regimens are different for NCFRB than those for CFRB.

Based on clinical common practice, the European Respiratory Society published three different pathways (numbered below) by which eradication may be attempted. Each step within any of these pathways is separated by sputum culture with escalation of treatment only should a patient remain culture positive.^1^

1. Prescribe an oral fluoroquinolone for 2 weeks. Failure is followed by an intravenous cell wall inhibitor combined with an aminoglycoside. Persistent culture positivity is treated with a daily inhaled antibiotic such as colistin, tobramycin, or gentamicin for a total treatment duration of 3 months.
2. Start with intravenous antimicrobial therapy (as delineated in pathway 1) for 2 weeks. Failure is followed by inhaled antimicrobial therapy (as described in pathway 1) for a total treatment duration of 3 months.
3. Initiate an inhaled antibiotic for 2 weeks combined with either an oral fluoroquinolone or IV antimicrobial therapy. Failure is followed by daily inhaled antibiotics for total treatment duration of 3 months.

#### Chronic pulmonary therapy

The specifics of ASL has not been as well examined in NCFRB as in CFRB. Also limiting the strength of evidence supporting osmotic and mucolytic therapy in the setting of NCFRB is the dearth of data and the paucity of subjects in available studies. Nevertheless, as there is evidence that 7% of HTS results in improved ppFEV_1_ and QOL scores, European Respiratory Society (ERS) guidelines recommend its use in this population.^1,85^ Use of inhaled mannitol is not clear in this population. Although inhaled mannitol increases 24-h sputum weight and time to first PEx, it does not decrease the frequency of PEx.^86^ Also, in a phase III trial that excluded patients with a positive mannitol provocation test, 1.8% of patients still experienced bronchospasm in the study arm, whereas 0% reported bronchospasm in the placebo group.^87^ Finally, studies utilizing inhaled mannitol and 6% HTS did not result in improved ppFEV_1._^86,88^ None of these therapies has been shown to decrease the rate of PEx.^1^

Symptomatic similarities between CFRB and NCFRB have led to the assumption that NCFRB is a parallel disease that should respond similarly to therapies proven effective in CFRB. Such a supposition has proven dangerous, such as the use of rhDNase in NCFRB patients, which is deleterious their FEV_1_ and was associated with an increase in PEx.^89^

#### Antimicrobial agents

Treatment with inhaled antibiotics is recommended for patients with NCFRB experiencing three or more exacerbations in the preceding year regardless of their pulmonary microbiota.^1,43^ They may be treated with an inhaled antibiotic, a macrolide, or both. Those chronically infected with *P. aeruginosa* begin with daily nebulized antibiotics (as there is no data as yet to support a monthly on–off cycle).^1^ These recommendations are based primarily on two randomized placebo-controlled studies demonstrating improved clinical outcomes in those prescribed suppressive inhaled antimicrobial therapy:

1. Colistin twice daily (Haworth and colleagues; decreased time to first PEx in the subgroup deemed adherent to intervention).^90^
2. Gentamycin twice daily (Murray and colleagues; decreased sputum bacterial load, time to first PEx and frequency of PEx. Patients were not blinded).^91^

Note: Inhaled aztreonam is not recommended due to decreased QOL scores and increased side-effects.^1,92^ Macrolides are recommended to replace, or be added, should there be a lack of response or intolerance to the inhaled antimicrobial therapy.^1^

Individuals not chronically infected with *P. aeruginosa* should be started on macrolide therapy based on the results of three prominent randomized double-blinded placebo-controlled studies: EMBRACE, BAT, and BLESS.^1,93–95^ Both EMBRACE and BAT reported a reduction in the frequency of PEx. BLESS had similar results, but this was manifested in those with *P. aeruginosa* infection. Despite this finding in BLESS, macrolide therapy is suggested as the first choice for frequent exacerbators without chronic *P. aeruginosa* infection. In this population, the ERS further suggests (although there is poor supporting evidence) that, should a patient continue to exacerbate or be macrolide intolerant, one may prescribe long-term targeted inhaled therapy. Should further clinical decline occur, a patient may be subsequently placed on a targeted oral antibiotic.^1^

It is important to remember that, prior to initiation of macrolide therapy, coinfection with NTM must be excluded.^1^ Additionally, continued screening for NTM ought to be performed throughout therapy along with regular monitoring for drug toxicity such a QT prolongation, ototoxicity, and nephrotoxicity.^1,39^

#### Anti-inflammatory medications

As in CFRB, inhaled corticosteroids are indicated for patients diagnosed with concomitant asthma or chronic obstructive pulmonary disease.^1^ HMG Co-A reductase inhibitors have not yet been found effective in patients with NCFRB.^1^ Ibuprofen has not yet been studied in NCFRB, and, thus, there are no recommendations regarding its use.^50^ Also, as in CFRB, leukotriene modifiers require further study before they are recommended for chronic use.^96^

#### Thoracic surgery

Surgical indications for NCFRB are broader than those for CFRB. Similarly to CF, treatment for localized refractory infection and uncontrolled hemoptysis are included.^97^ Refractory infection in this population is very often due to NTM.^98,99^ Many of these patients, however, often experience a significantly diminished QOL even with ppFEV_1_ approaching 70%.^100^ Thus, in contrast to CF, diminished QOL may be a prompt for surgical evaluation.^100^ Additionally, as many cases of NCFRB are due to localized structural change such as right middle lobe syndrome or postinfectious bronchiectasis (such as prior tuberculosis) resection may result in symptom resolution or improvement.^97,101^ Zhang and colleagues retrospectively reviewed the outcomes of 790 patients who underwent resection for localized disease, significant hemoptysis, pulmonary abscess, empyema, chronic productive cough, chronic infection refractory to antimicrobial therapy.^99^ They reported symptom resolution in 60.5%, improvement in 14.1%, and no change or decline in 14.8% of patients. Other results emphasized that meticulous patient selection is paramount for successful outcome as age > 70 years and renal failure (creatinine clearance < 60 ml/min) were both associated with increased postoperative mortality. Additionally, pre-operative ppFEV_1_ < 50% was associated with increased risk of postoperative complications.^100^ Thus, following careful patient selection, surgical consideration may occur more commonly than in CFRB.^99,100^

## Conclusion

Infection control and prevention is paramount in the care of patients with both CFRB and NCFRB. It is a complex endeavor combining education, protocol development, environmental changes, behavioral changes and adherence to treatment plans, which, when combined, decrease bacterial transmission and temper the pulmonary consequences of bacterial infection. Detailed attention from a team of individuals committed to its implementation can prevent or delay the acquisition of many pathologic organisms. Further, by controlling the effects of chronic infection we can offer our patients decreased mortality, fewer exacerbations and, most importantly, improved QOL.
